# The genetic insulator RiboJ increases expression of insulated genes

**DOI:** 10.1186/s13036-018-0115-6

**Published:** 2018-10-29

**Authors:** Kalen P. Clifton, Ethan M. Jones, Sudip Paudel, John P. Marken, Callan E. Monette, Andrew D. Halleran, Lidia Epp, Margaret S. Saha

**Affiliations:** 1 Department of Biology, Integrated Science Center, The College of William and Mary, 540 Landrum Drive, Williamsburg, VA 23185 USA; 2 Division of Biology and Bioengineering, California Institute of Technology, Pasadena, CA 91125 USA

**Keywords:** RiboJ, Insulation, Characterization, Digital droplet PCR, Genetic circuit, Ribozyme

## Abstract

A primary objective of synthetic biology is the construction of genetic circuits with behaviors that can be predicted based on the properties of the constituent genetic parts from which they are built. However a significant issue in the construction of synthetic genetic circuits is a phenomenon known as context dependence in which the behavior of a given part changes depending on the choice of adjacent or nearby parts. Interactions between parts compromise the modularity of the circuit, impeding the implementation of predictable genetic constructs. To address this issue, investigators have devised genetic insulators that prevent these unintended context-dependent interactions between neighboring parts. One of the most commonly used insulators in bacterial systems is the self-cleaving ribozyme RiboJ. Despite its utility as an insulator, there has been no systematic quantitative assessment of the effect of RiboJ on the expression level of downstream genetic parts. Here, we characterized the impact of insulation with RiboJ on expression of a reporter gene driven by a promoter from a library of 24 frequently employed constitutive promoters in an *Escherichia coli* model system. We show that, depending on the strength of the promoter, insulation with RiboJ increased protein abundance between twofold and tenfold and increased transcript abundance by an average of twofold. This result demonstrates that genetic insulators in *E. coli* can impact the expression of downstream genes, information that is essential for the design of predictable genetic circuits and constructs.

**Electronic supplementary material:**

The online version of this article (10.1186/s13036-018-0115-6) contains supplementary material, which is available to authorized users.

## Background

A fundamental goal of synthetic biology is the construction of genetic circuits that can perform a variety of useful functions that include environmental sensing and remediation, metabolic engineering, pharmaceutical and fuel production, drug delivery, and even cellular computation [1]. Similar to other engineering disciplines, a guiding tenet of circuit design is the use of well-characterized, modular parts—promoters, ribosome binding sites, coding regions—which in turn can be combined to produce a construct that behaves in a consistent, predictable manner [2]. This predictability relies on the use of parts whose behaviors are unaffected by other parts in the construct. However, despite careful characterization, constituent parts frequently do not behave in a predictable manner; rather, they are influenced by their particular genetic context, that is their neighboring sequences [3].

**Fig. 1:**
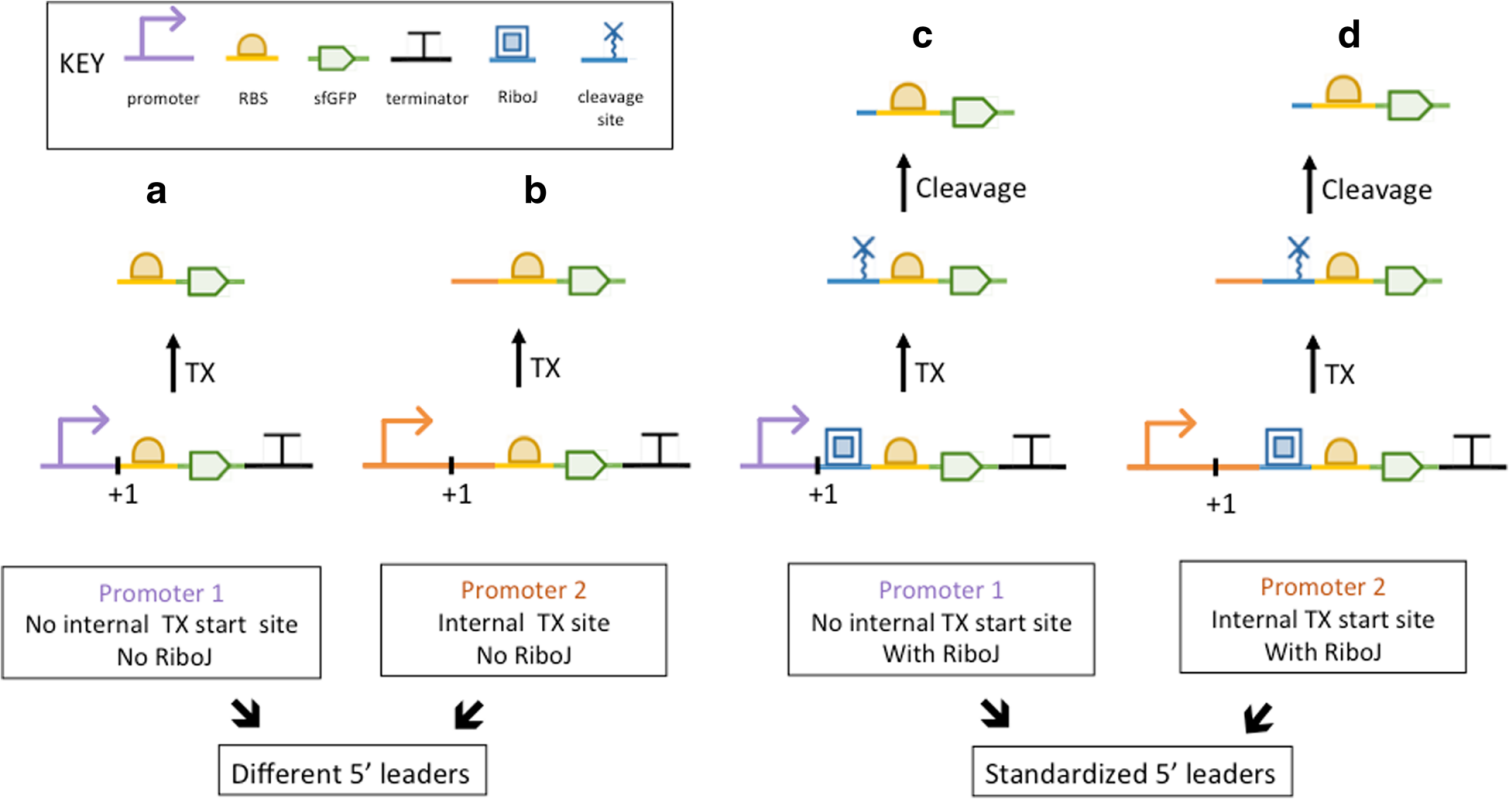
Schematic depicting role of RiboJ insulation on transcripts. Constructs in (**a**) and (**b**) have same coding region and are identical at the DNA level except for different promoters. However, the constructs in (**a**) and (**b**) result in different transcripts. Construct (**b**) has a synthetic promoter that contains an internal transcriptional start site leading to the inclusion of additional sequences in the transcript. This 5’ RNA leader can affect the stability of the RNA and result in different translational expression properties as well. The same constructs depicted in (**a**) and (**b**) are shown in (**c)** and **d**, except with the addition of the insulator RiboJ. Following transcription, the ribozyme RiboJ self-cleaves, resulting in standardized and identical transcripts

One significant source of the effect of genetic context is the use of synthetic promoters containing regulatory sequences downstream of the transcriptional start site. This additional sequence is transcribed, leading to the inclusion of unintended nucleotides termed “RNA leaders” at the 5′ end of the transcript. It has been shown that these RNA leaders can modify the stability and secondary structure of mRNA, which in turn alters the translational properties of genetic constructs [4]. The nature of these alterations is specified by the interactions between a given RNA leader and the downstream sequence of the transcript. Thus, changes to a construct’s behavior will depend on both the specific promoter used and downstream composition of a construct (Fig. 1).

To circumvent the effects of unintended RNA leaders, constructs can be designed to include genetic insulators, which isolate parts from unwanted interactions with their neighboring regions. One routinely used genetic insulator is the synthetic self-cleaving ribozyme RiboJ. The utility of RiboJ as an insulator was first demonstrated by Lou et al. [4]. These investigators sought to characterize the transfer function of a NOT gate, in which the activity of an input promoter repressed the activity of an output promoter. When the circuit was driven by different inducible promoters, they found an unanticipated promoter-dependent effect, in which variable RNA leaders prevented the input-output responses from being quantitatively identical. The use of RiboJ led to equivalent transfer functions regardless of input promoter, thereby insulating the circuit from the promoter-dependent effect.

RiboJ is a 75 nucleotide sequence consisting of a satellite RNA of tobacco ringspot virus (sTRSV) derived ribozyme followed by a 23 nucleotide hairpin. When used as an insulator, RiboJ is inserted in a construct at the junction of a promoter and its downstream sequence. During post-transcriptional processing, the ribozyme self-cleaves, removing upstream sequences and therefore eliminating promoter-associated RNA leaders. After cleavage, only a hairpin-containing sequence from the uncleaved region of RiboJ remains upstream of the ribosome binding site and the gene of interest. As a result of this processing, the 5′ end of every insulated gene will be identical, regardless of the choice of promoter. Therefore, insulation with RiboJ should serve to standardize the behavior of promoters across constructs, aiding the design of predictable genetic constructs.

Although RiboJ is frequently used as an insulator in genetic constructs, there has been limited characterization of what effect, if any, this insulation has on the expression level of downstream genetic parts. Since the construction of accurate and predictable genetic circuits could be impeded if insulation with RiboJ has any unanticipated effects on gene expression levels, we characterized the impact of RiboJ insulation on the gene expression of a set of constitutive promoter constructs. In this paper, we report that insulation with RiboJ increases gene expression levels at both the RNA and protein level across a wide range of promoter strengths.

## Methods

### Construction of library

For each promoter we created two measurement constructs. The first contained the promoter, the ribosome binding site (RBS) Bba_B0034, superfolder green fluorescent protein (sfGFP) [4], and the double terminator Bba_B0015. The second differed from the first only by the presence of RiboJ immediately upstream of the RBS sequence. Each of these 48 constructs and a negative control (J23101 B0034 LacI B0015) were cloned from linear gene fragments onto the low copy plasmid backbone pSB3K3 using NEB HiFi DNA assembly. Linear sequences for the promoters were obtained via PCR overlap of primers from IDT, and the remaining fragments were obtained from a template construct created from IDT gBlocks. Construct were transformed in 5-alpha *Escherichia coli* (*E. coli)* (NEB) and isolated via miniprep (NEB Monarch miniprep), and confirmed by Sanger sequencing.

### Quantification of protein expression

For assessment of RiboJ’s impact on expression level, each construct was transformed into chemically competent BL21 *E.Coli* (NEB) using the manufacturer’s protocol. Then three distinct colonies were confirmed by colony PCR and grown overnight in 3 mL of LB containing 16 μg/mL kanamycin. After 12–14 h, saturated cultures were diluted 1:100 into 3 mL of M9 media containing 0.4% glucose and 16 μg/mL kanamycin and were grown to an Optical Density at 600 nm (OD600) of 0.5 as measured by plate reader (Biotek Synergy H1). For each sample, 1.5 mL of culture was pelleted at 6000x rpm, resuspended in 0.5 mL of Trizol, and stored at -20C immediately and then later moved to -80C for later RNA extraction. Of the remaining culture, 50 μl was filtered with 20 μm filters (CellTrics) into 0.5 mL of PBS and sfGFP expression was measured by flow cytometry for at least 10,000 cells per sample on the FL1 channel of a Bio-Rad S3e cell sorter. Absolute fluorescence for each sample was calibrated using Spherotech Rainbow Calibration beads and the python package FlowCal [5].

### RNA isolation and quantification

Samples in Trizol were thawed on ice and homogenized using Lysing Matrix B (MP Biomedical) along with a Bead Ruptor (Omni) for 1 min at speed 6, and the total RNA of each sample was isolated using the MagMAX™ mirVana™ Total RNA Isolation Kit (Applied Biosystems), with a 20 min DNAse step using Turbo DNase enclosed with the kit. DNase activity was halted by the addition of 200 mM EDTA, and RNA was repurified using the same MagMAX kit as before, quantified via a Nanodrop Spectrophotometer, and stored in aliquots at -80C. Following the manufacturer’s protocol, 500 ng of total RNA was reverse transcribed into cDNA using an iScript cDNA Synthesis Kit (Bio-Rad) and quantified via Nanodrop. Then Uroporphyrinogen-III C-methyltransferase (CysG) [6] and sfGFP transcript levels were measured separately via Taqman Assay (Thermofisher) using 1.0 or 0.1 ng of cDNA in a 20 μL volume reverse transcription digital droplet qPCR (ddPCR) (Bio-Rad) reaction. Positive droplet thresholds were set at 2800 for CysG and 4750 for sfGFP, and for each sample a no reverse transcriptase (RT) control was run with both assays; each plate also contained a no template control.

### Analysis methods

We used Flowcal to report fluorescence in Molecules of Equivalent Fluorophore (MEF) instead of arbitrary units. Provided the fluorescence of Spherotech Rainbow Calibration beads, Flowcal is able to determine the geometric mean of absolute fluorescence in MEF of at least 10,000 cells for each of our samples. Furthermore, we normalized the absolute fluorescence measured for our reporter constructs by subtracting the absolute fluorescence of the negative control construct. All calculations were subsequently done with the normalized absolute fluorescence. The data and calculations are provided in Additional File 1.

We created two (with RiboJ and without RiboJ) measurement constructs for each promoter and each of these two constructs had three biological replicates for which we measured fluorescence and transcript abundance. Therefore each promoter has six protein and RNA measurements, three with RiboJ and three without RiboJ. We utilized two methods to represent the fold-change associated with RiboJ for each promoter. In the first method, the fold-change is a single value that is calculated as a ratio of the geometric mean of the three replicates with RiboJ and the geometric mean of the three replicates without RiboJ. This first method, referred to as “fold change of means,” is equivalent to $\frac{\sqrt[3]{y_1{y}_2{y}_3}}{\sqrt[3]{x_1{x}_2{x}_3}},$where for a given promoter *x*_*k*_ is the measurement of the *k*th replicate without RiboJ and *y*_*k*_ is the measurement of the *k*th replicate with RiboJ. Average fold-changes and ranges of fold-changes reported are determined using “fold change of means.” In the second method, the fold-change is represented by a set of nine values that correspond to all pairwise fold changes between replicates. These nine values are all possible ratios given by dividing a measurement of one of the replicates with RiboJ by a measurement of one of the replicates without RiboJ. This second method, referred to “pairwise fold-changes,” is summarized as $\left\{\frac{y_i}{x_j}:i\in \left\{1,2,3\right\},j\in \left\{1,2,3\right\}\right\}$, where *x*_*k*_ and *y*_*k*_ are defined the same as above.

Additionally, we determined the distributions of fold-changes that are not associated with insulation with RiboJ for measurements of fluorescence and sfGFP transcript abundance. This null distribution captures changes that would be introduced by natural expression variance and intrinsic noise in the measurement techniques. For each promoter, these null pairwise fold-changes are computed by $\left\{\frac{y_i}{y_j},\frac{x_i}{x_j}:i\in \left\{1,2,3\right\},j\in \left\{1,2,3\right\},i\ne j\right\}$, that is dividing replicates from the same RiboJ condition and excluding identity ratios, where a replicate is divided by itself.

## Results

We assembled two sets of reporter constructs that differed only by the presence RiboJ. Each set contained 24 constructs, which each expressed an sfGFP reporter with a different synthetic *E. coli* constitutive promoter part. The collection of promoter parts spans a wide range of transcriptional strengths and includes the well characterized and commonly used Anderson Promoter Library [7], as well as synthetic hybrid promoters such as R0011 (PLlacO-1) **(**Additional File 2: Table S1). Each construct was transformed into BL21 *E. coli* and expression of the fluorescent reporter was measured using flow cytometry [5].

**Fig. 2:**
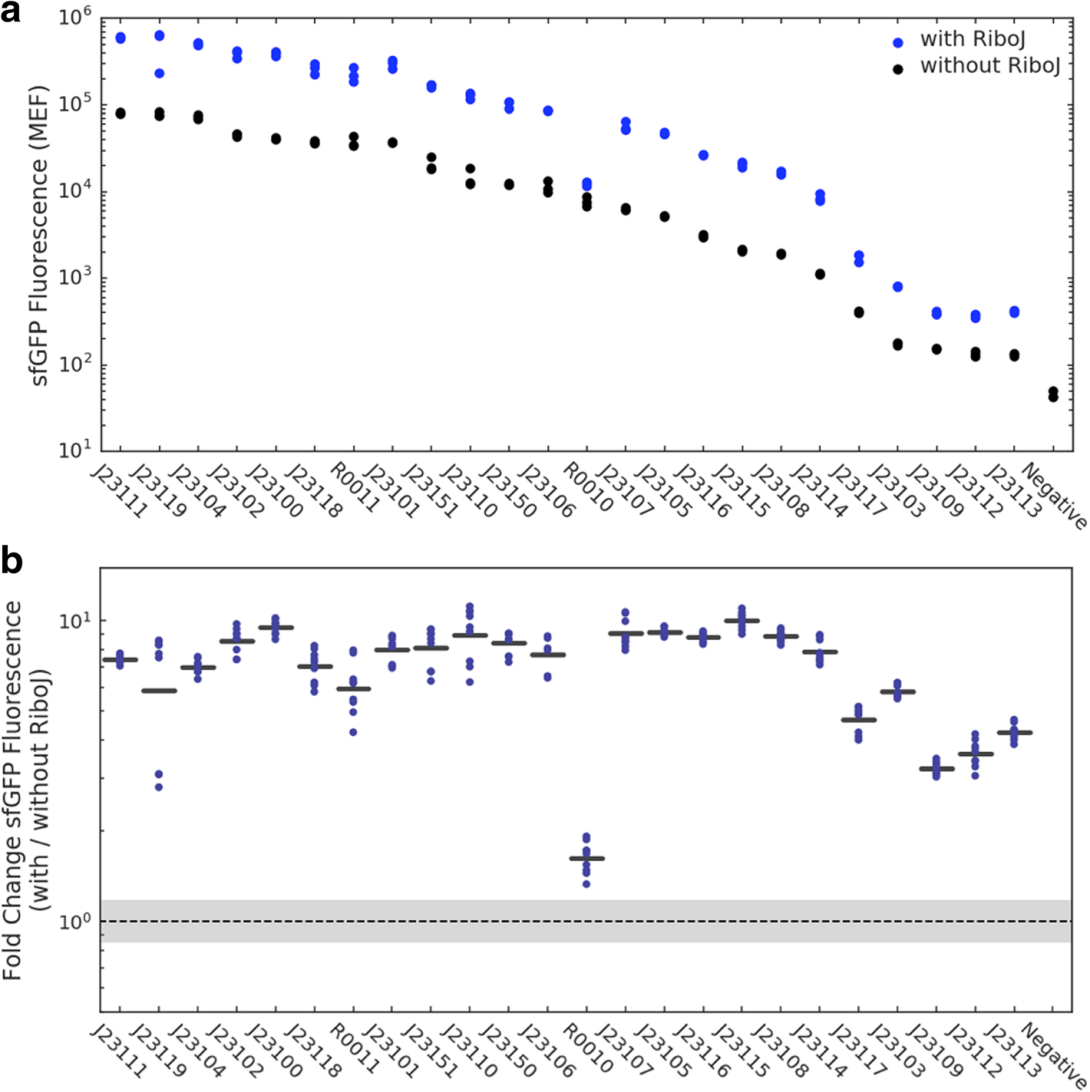
**a** Absolute fluorescence of constructs denoted by BioBrick ID (Additional File 2: Table S1), with (blue) and without (black) RiboJ insulation as measured by calibrated flow cytometry. Each dot represents the geometric mean fluorescence of *n* > 10,000 cells. **b** Fold change in fluorescence of constructs when insulated with RiboJ. Bars represent the fold change in the mean fluorescence across replicates, and dots represent all pairwise fold changes between replicates. The dashed line and grey region indicate one geometric SD factor around the geometric mean of a null fold change distribution computed from the fluorescence data

We found that for each pair of promoter parts, the construct insulated by RiboJ had greater absolute fluorescence than the corresponding construct without RiboJ **(**Fig. 2a**,** Additional File 2: Figure S1), with increases ranging from twofold to tenfold **(**Fig. 2b**)**. We found that the fold change in expression with RiboJ appears to exhibit bimodality between “strong” and “weak” promoters. For the “strong” group, composed of constructs driven by 18 of our assay’s 19 strongest promoters, insulation with RiboJ increased absolute fluorescence by an average of eightfold. Within this group, effects ranged from a sixfold to a tenfold increase in fluorescence. For the remaining “weak” promoters, we found that the magnitude of the increase was lower, with an average increase of fourfold, ranging from twofold to sixfold. While we found that RiboJ exhibited bimodality between “stronger” and “weaker” promoters, we did not find a continuous monotonic relationship between promoter strength and fold change in protein expression **(**Additional File 2: Figure S2).

**Fig. 3:**
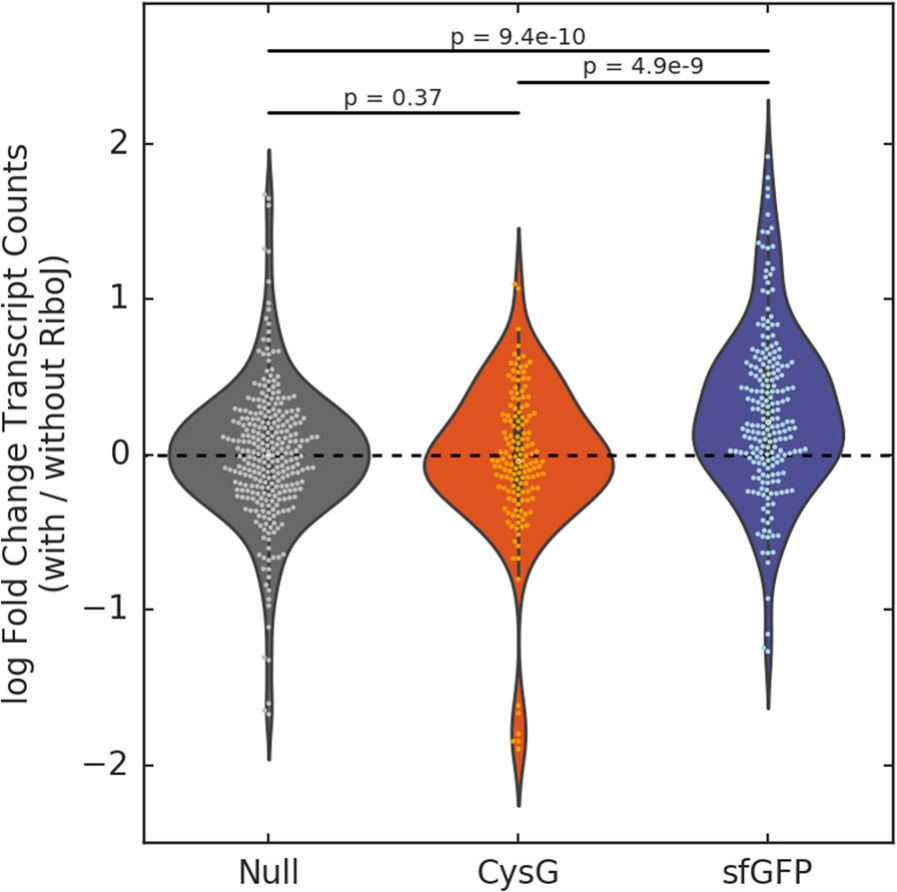
Fold change in the transcript abundance of CysG, sfGFP, and null distribution when promoter constructs are insulated with RiboJ. *P* values were calculated using Welch’s one-tailed *t*-tests with hypotheses sfGFP > Null (*p* = 9.41e-10) and sfGFP > CysG (*p* = 4.94e-09). For the comparison of Null and CysG, Welch’s two-tailed *t*-test was used (*p* = 0.37). Dots represent all pairwise ratios of replicates (9 dots per promoter)

Since the increase in protein expression with RiboJ could be attributed to either differential transcription or translation of insulated genes, we characterized the effect of insulation with RiboJ on the relative abundances of sfGFP transcripts. We used reverse transcription ddPCR with CysG serving as an endogenous reference [6]. We found that insulation of constructs with RiboJ increased sfGFP transcript abundance by an average of twofold, while there was no change in transcript abundance of our reference gene CysG on average **(**Additional File 2: Figs. S3, S4). The mean fold change for sfGFP transcript counts was greater than for a null distribution and for the endogenous reference gene, which indicates that the observed increase in the transcript abundance of sfGFP is indeed due to insulation with RiboJ **(**Fig. 3**,** Additional File 2: Fig. S5).

## Discussion

**Fig. 4:**
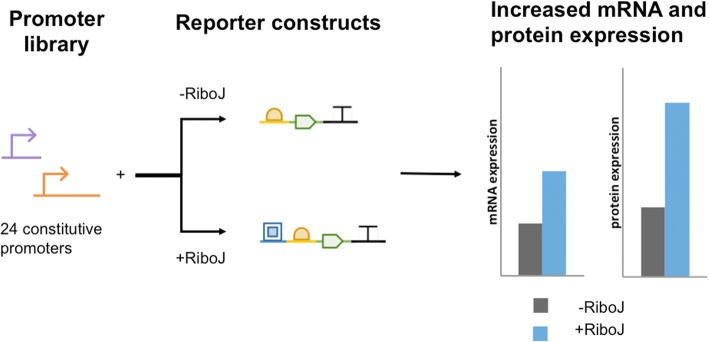
RiboJ increases gene expression levels. To characterize the impact of insulation with RiboJ on gene expression, a library of 24 frequently employed constitutive promoters was used to drive the expression of a sfGFP reporter construct. For each of the 24 promoters, we assembled two sets of reporter constructs that differed only by the presence RiboJ. Insulation with RiboJ increased mRNA expression by an average of twofold and increased protein expression between twofold and tenfold

The genetic insulator RiboJ is a valuable tool that aids the implementation of predictable genetic circuits by allowing promoter characterization to be standardized across genetic constructs. While insulation with RiboJ is widely used, there has been no comprehensive characterization of its effects on the expression of insulated genes. Here we provide a quantitative characterization of the effect of insulation with RiboJ on a collection of promoter constructs. We determined that insulating a construct with RiboJ leads to an increase in protein expression and transcript abundance **(**Fig. 4**)**; these increases may lead to inaccurate experimental fluorescence results when RiboJ is used.

Increased expression of both protein and mRNA in genes insulated by RiboJ could be explained by higher transcript abundance, which itself could result from RiboJ increasing the mRNA stability. Increased mRNA stability would lead to slower degradation of transcripts, leading to an increase in transcript abundance. This increased transcript abundance would then be amplified by translation, leading to greater protein expression levels. Potentially, this increase in mRNA stability could be due to the fact that, post-cleavage, the remaining RiboJ sequence forms a terminal hairpin on the transcript, which has been found to increase mRNA stability in other systems [4, 8].

In support of this explanation, we found that sfGFP transcript abundance was well correlated to sfGFP protein concentration **(**Additional File 2: Figure S6), which could imply that increased protein abundance is driven by a RiboJ-associated increase in mRNA abundance. However, we found that the fold change in transcript abundance for each construct did not correlate well with fold change in protein **(**Additional File 2: Figure S7), which suggests that increases in protein abundance due to RiboJ could be due to translational processes as well as increases in transcript abundance. An avenue by which RiboJ can affect translation of the transcript is via the hairpin at the end of the RiboJ sequence, downstream of the ribozyme. It has been suggested that this appended hairpin aids in exposing the RBS [4].

Further work is needed in order to gain an understanding of this phenomenon and to determine whether it is possible to design a ribozyme-based insulator which insulates effectively without modifying associated gene expression. Since hairpins form in the secondary structure of the ribozymes that have been used in the design of insulators [4], further engineering is necessary to ensure that this structural modification does not unpredictably affect circuit response due to altered RNA stability. Other methods for genetic insulation, such as the CRISPR-based insulation system pioneered by Qi and colleagues [9], may show promise for insulation properties that are independent of circuit output amplitude. Additional investigation is required to determine ways to manage the increases in gene expression under insulated conditions. Here we have only identified the increases, but since unanticipated increases in gene expression can decrease circuit performance by increasing cellular metabolic strain or by causing decreases in the dynamic range of portions of the circuit [10], this characterization provides valuable information for the design and implementation of genetic constructs and circuits.

## Additional files

Additional file 1: **Data.csv** Experimental data. Analysis.ipynb Analysis methods as a jupyter notebook. (ZIP 16 kb)

Additional file 2: **Figure S1.** Fold Change in sfGFP Fluorescence associated with RiboJ Insulation. **Figure S2.** RiboJ-associated fold change does not strongly monotonically correlate with expression strength. **Figure S3.** Counts and fold change for sfGFP transcripts. **Figure S4.** Counts and fold change for CysG transcripts. **Figure S5.** RiboJ-associated fold change of mean transcript counts across replicates. **Figure S6.** sfGFP fluorescence correlates with sfGFP transcript counts. **Figure S7.** sfGFP fluorescence fold change is generally higher than sfGFP transcript count fold change. **Table S1.** Promoter sequences ordered by BioBrick ID. **Construct design.** Sequences of constructs with and without RiboJ. (PDF 618 kb)

## Abbreviations

CysG: Uroporphyrinogen-III C-methyltransferase
ddPCR: Digital droplet qPCR
*E. coli*: *Escherichia coli*
MEF: Molecules of Equivalent Fluorophore
RBS: Ribosome binding site
sfGFP: Superfolder green fluorescent protein
sTRSV: Satellite RNA of tobacco ringspot virus
