# Supplementary material for: The genetic insulator RiboJ increases expression of insulated genes
Source: J Biol Eng. 2018 Oct 29;12:23. doi: 10.1186/s13036-018-0115-6 (PMC6206723; doi:10.1186/s13036-018-0115-6)
Supplement: Supplementary file 2 — Figure S1. Fold Change in sfGFP Fluorescence associated with RiboJ Insulation. Figure S2. RiboJ-associated fold change does not strongly monotonically correlate with expression strength. Figure S3. Counts and fold change for sfGFP transcripts. Figure S4. Counts and fold change for CysG transcripts. Figure S5. RiboJ-associated fold change of mean transcript counts across replicates. Figure S6. sfGFP fluorescence correlates with sfGFP transcript counts. Figure S7. sfGFP fluorescence fold change is generally higher than sfGFP transcript count fold change. Table S1. Promoter sequences ordered by BioBrick ID. Construct design. Sequences of constructs with and without RiboJ. (PDF 618 kb) [file 13036_2018_115_MOESM2_ESM.pdf]

The Genetic Insulator RiboJ Increases Expression of Insulated Genes

Authors: Kalen P Clifton[#1], Ethan M Jones[#1], Sudip Paudel[1], John P Marken[2], Callan E Monette[1], Andrew D Halleran[2], Lidia Epp[1], Margaret S Saha[1*]
#These authors equally contributed to the work
*Corresponding Author

Contents: Figures S1-7, Table S1, Construct design

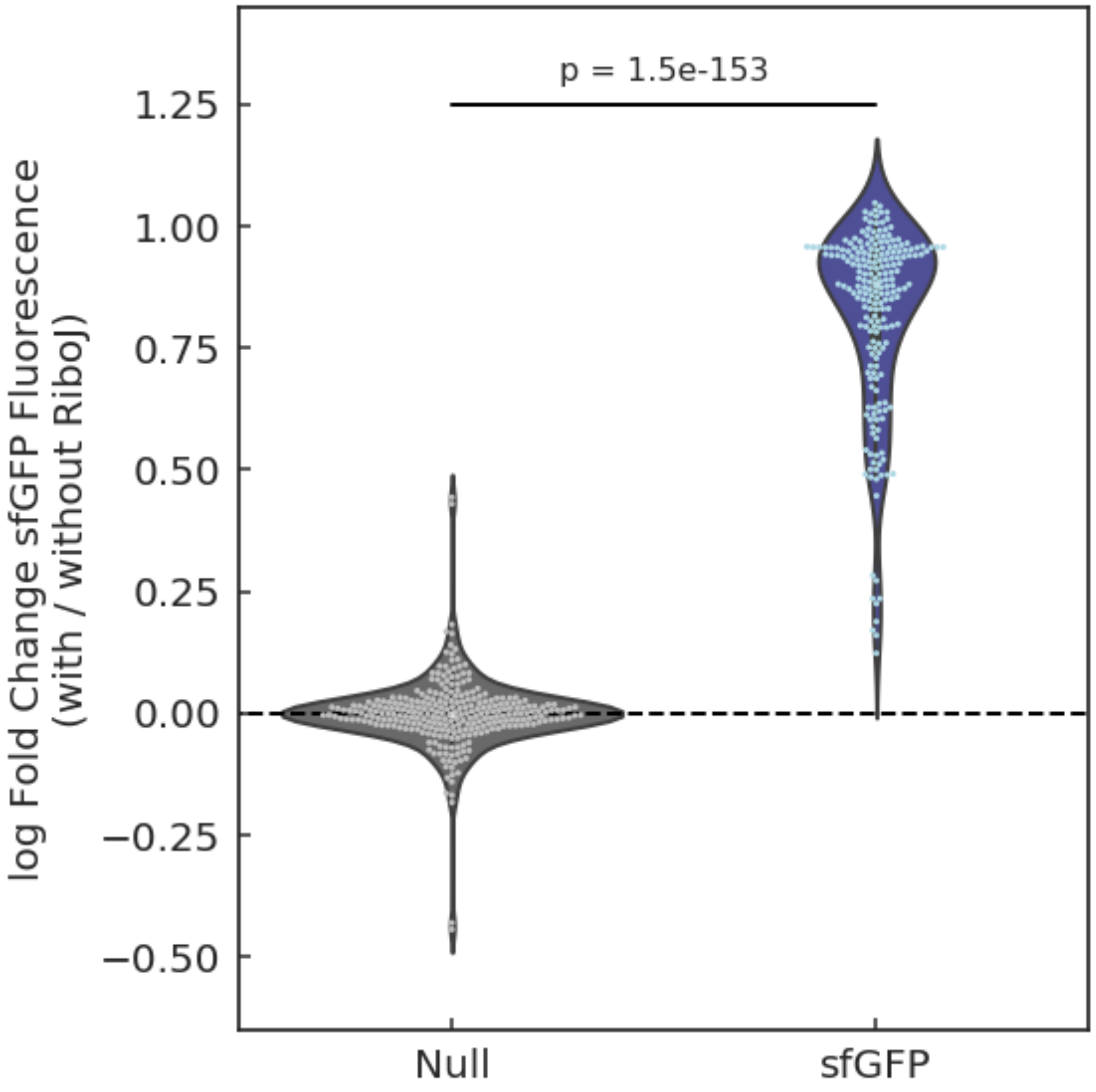

Figure S1: Fold Change in sfGFP Fluorescence associated with RiboJ Insulation.
Dots indicate the pairwise fold change values computed between all replicates of a given construct. All constructs are pooled together into a single distribution. The null fold change distribution was computed from the sfGFP fluorescence data (Supplemental Methods). P-value was calculated from Welch's one-tailed t-test with hypothesis sfGFP > Null (p=1.5e-153).

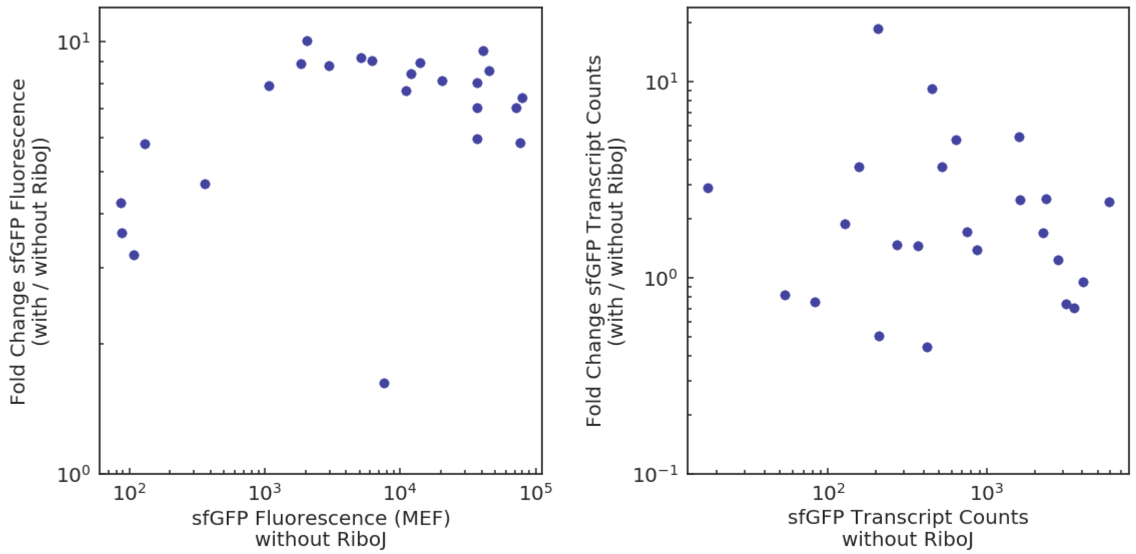

Figure S2: RiboJ-associated fold change does not strongly monotonically correlate with expression strength.
Spearman's rho = 0.24 (p = 0.26) for the relationship between uninsulated sfGFP fluorescence and RiboJ-associated fold change in sfGFP fluorescence (left), so we cannot claim that there is a monotonic correlation between these variables. Spearman's rho = -0.11 (p = 0.60) for the relationship between the uninsulated sfGFP transcript counts and RiboJ-associated fold change in sfGFP transcript counts (right), so we cannot claim that there is a monotonic correlation between these variables.

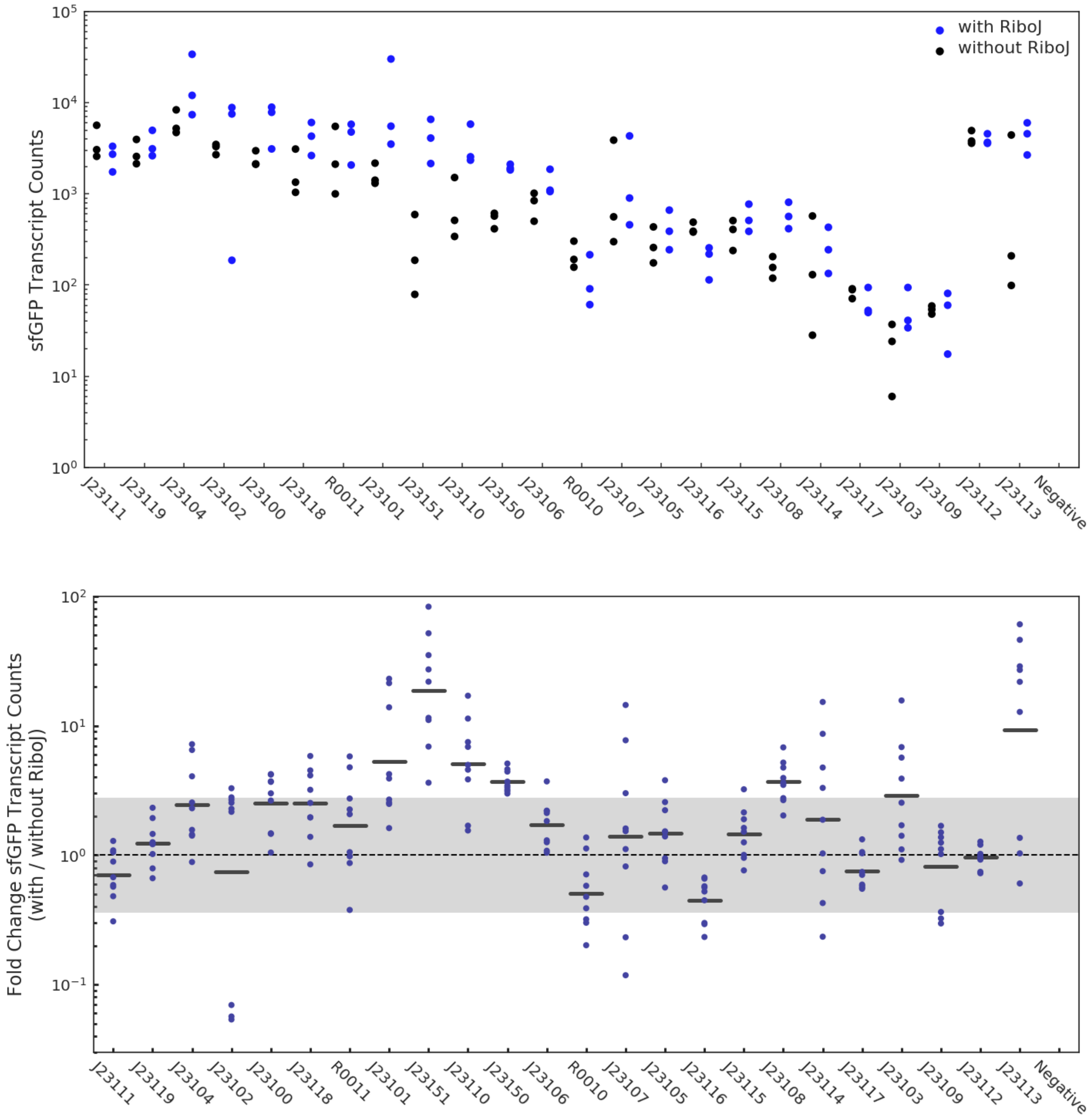

Figure S3: Counts and fold change for sfGFP transcripts.
Top: sfGFP transcript counts for each biological replicate of each construct, obtained by ddPCR. Transcript count values for the negative control were <1.
Bottom: RiboJ-associated fold change in sfGFP transcript count values. Black bars represent the fold change in the mean transcript count across replicates, and dots represent all pairwise fold changes between replicates. The grey region and dashed line indicate one geometric SD factor around the geometric mean of the null fold change distribution computed from the sfGFP transcript count data (Supplemental Methods).

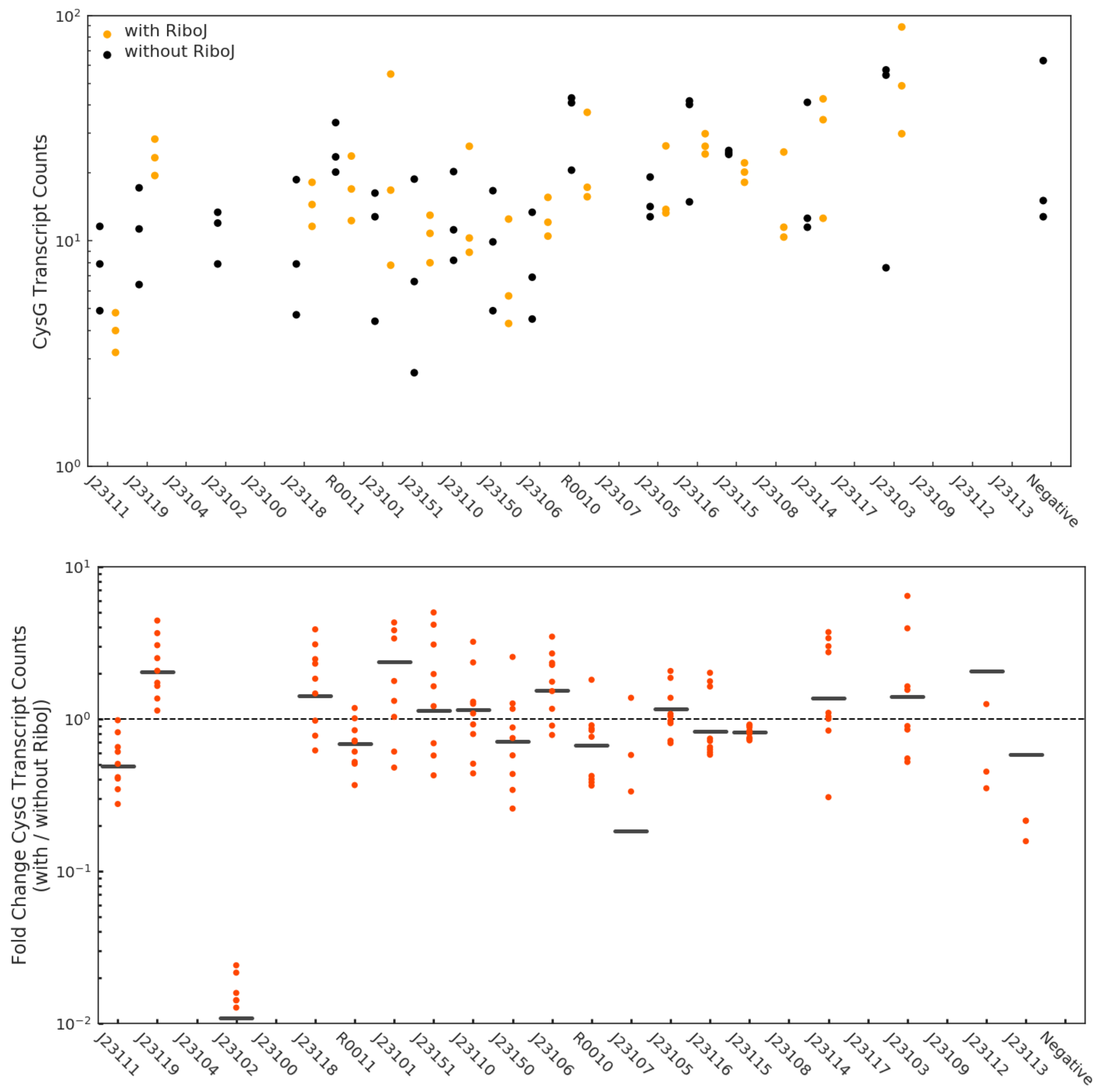

Figure S4: Counts and fold change for CysG transcripts.
Top: CysG transcript counts for each biological replicate of each construct, obtained ddPCR. Transcript count values that were less than 1 are not shown.
Bottom: RiboJ-associated fold change in CysG transcript count values. Black bars represent the fold change in the mean transcript count across replicates, and dots represent all pairwise fold changes between replicates. Transcript count values <1 were excluded from fold change calculations.

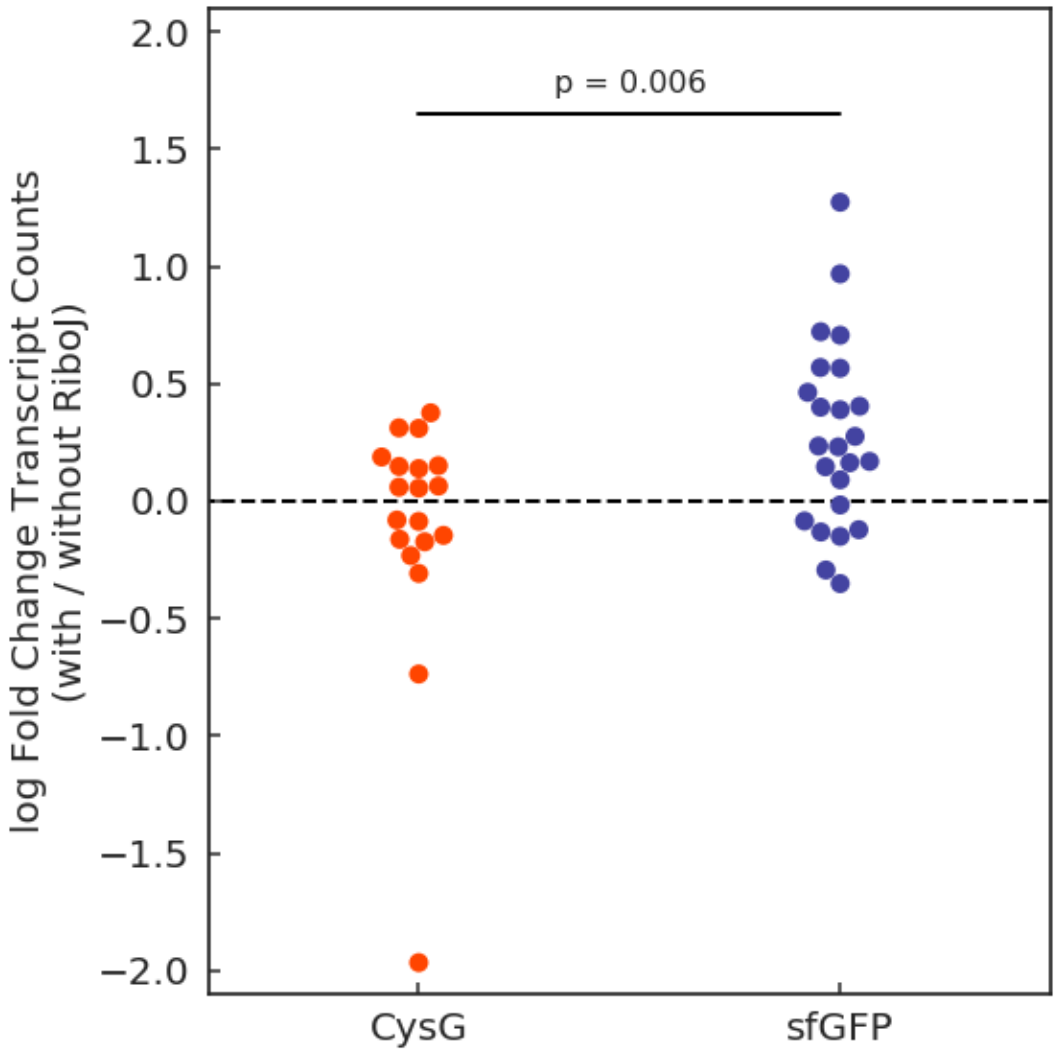

Figure S5: RiboJ-associated fold change of mean transcript counts across replicates. *Fold change in the transcript abundance of CysG and sfGFP when promoter constructs are insulated with RiboJ. Dots depict the fold change in the mean transcript count across the three replicates for a given construct (Supplemental Methods). All constructs are pooled into a single distribution. P-value was calculated from Welch's one-tailed t-test with hypothesis sfGFP > CysG (p=0.006).*

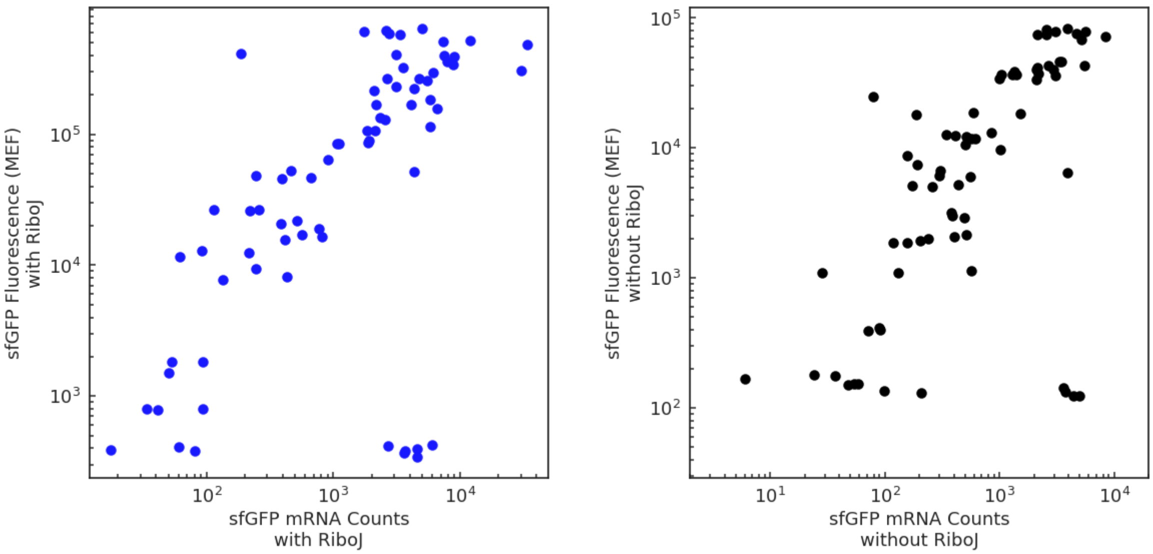

Figure S6: sfGFP fluorescence correlates with sfGFP transcript counts.
Each dot depicts the relationship between a replicate fluorescence measurement and a replicate transcript count measurement, so each construct will appear 9 times on a given plot. Spearman's rho = 0.61 (p = 1.8e-8) for the transcript count-fluorescence correlation in the RiboJ-insulated constructs (left), and Spearman's rho = 0.67 (p = 4.3e-11) for the transcript count-fluorescence correlation in the non-insulated constructs (right).

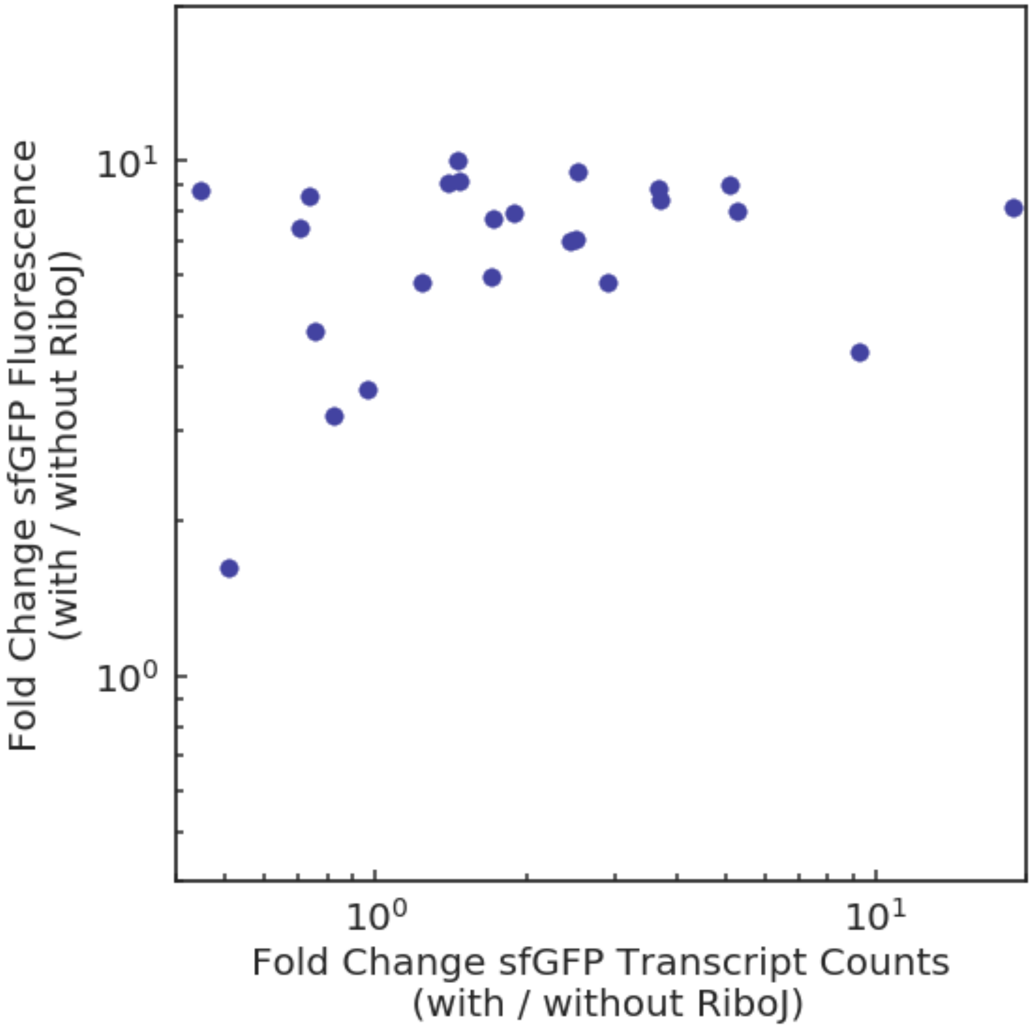

Figure S7: sfGFP fluorescence fold change is generally higher than sfGFP transcript count fold change.
Each dot depicts the relationship between the fold change in the geometric mean fluorescence and the fold change in mean transcript counts associated with RiboJ across all replicates for a given construct. For all but two promoters, the fold change in fluorescence is higher than the fold change in transcript count. As Spearman's rho = 0.21 (p = 0.32), we cannot claim that there is a monotonic correlation between the variables.

Table S1 (Promoter sequences)

**J23100**	**ttgacggctagctcagtcctaggtacagtgctagc**
**J23101**	**tttacagctagctcagtcctaggtattatgctagc**
**J23102**	**ttgacagctagctcagtcctaggtactgtgctagc**
**J23103**	**ctgatagctagctcagtcctagggattatgctagc**
**J23104**	**ttgacagctagctcagtcctaggtattgtgctagc**
**J23105**	**tttacggctagctcagtcctaggtactatgctagc**
**J23106**	**tttacggctagctcagtcctaggtatagtgctagc**
**J23107**	**tttacggctagctcagccctaggtattatgctagc**
**J23108**	**ctgacagctagctcagtcctaggtataatgctagc**
**J23109**	**tttacagctagctcagtcctagggactgtgctagc**
**J23110**	**tttacggctagctcagtcctaggtacaatgctagc**
**J23111**	**ttgacggctagctcagtcctaggtatagtgctagc**
**J23112**	**ctgatagctagctcagtcctagggattatgctagc**
**J23113**	**ctgatggctagctcagtcctagggattatgctagc**
**J23114**	**tttatggctagctcagtcctaggtacaatgctagc**
**J23115**	**tttatagctagctcagcccttggtacaatgctagc**

**J23116**	**ttgacagctagctcagtcctagggactatgctagc**
**J23117**	**ttgacagctagctcagtcctagggattgtgctagc**
**J23118**	**ttgacggctagctcagtcctaggtattgtgctagc**
**J23119**	**ttgacagctagctcagtcctaggtataatgctagc**
**J23150**	**tttacggctagctcagtcctaggtattatgctagc**
**J23151**	**ttgatggctagctcagtcctaggtacaatgctagc**
**R0010**	**caatacgcaaaccgcctctccccgcgcgttggccgattcattaatgcagctggcacgacaggtttcccgactggaaagcgggcagtgagcgcaacgcaat**
**R0011**	**aattgtgagcggataacaattgacattgtgagcggataacaagatactgagcaca**

Construct Design:
Each construct of the two constructs below was assembled with each of the 24 promoter sequence (See supplementary sequences) at the site labeled xxx.

RiboJ Construct
>Promoter Part
XXXXXXXXXXXXXXXXXXXXXXXXXXXXXXXXXXXXXXXX

>RiboJ (from Lou *et al.* Supplement section V)
Agctgtcaccggatgtgctttccggtctgatgagtccgtgaggacgaaacagcctctacaaataattttgtttaa

>BioBrick Scar (from Lou *et al.* Supplement section V)
ACTAGA

> B0034 w/ Spacer (from Lou *et al.* Supplement section V)
AAAGAGGAGAAATACTAG

>sfGFP (modified from Lou *et al.* Supplement section V)
Atgcgtaaaggcgaagagctgttcactggtgtcgtccctattctggtggaactggatggtgatgtcaacggtcataagttttccgtgcgtg
gcgagggtgaaggtgacgcaactaatggtaaactgacgctgaagttcatctgtactactggtaaactgccggtaccttggccgactctg
gtaacgacgctgacttatggtgttcagtgctttgctcgttatccggaccatatgaagcagcatgacttcttcaagtccgccatgccggaag
gctatgtgcaggaacgcacgatttcctttaaggatgacggcacgtacaaaacgcgtgcggaagtgaaatttgaaggcgataccctgg
taaaccgcattgagctgaaaggcattgactttaaagaagacggcaatatcctgggccataagctggaatacaattttaacagccaca
atgtgtacattaccgcagataaacaaaaaaatggcattaaagcgaatttcaaaattcgccacaacgtggaggatggcagcgtgcag
ctggctgatcactaccagcaaaacactccaatcggtgatggtcctgttctgctgccagacaatcactatctgagcacgcaaagcgttct
gtctaaagatccgaacgagaaacgcgatcatatggttctgctggagttcgtaaccgcagcgggcatcacgcatggtatggatgaact
gtacaaatgatga

>BBa_B0015 Part-only sequence – double terminator
ccaggcatcaaataaaacgaaaggctcagtcgaaagactgggcctttcgttttatctgttgtttgtcggtgaacgctctctactagagtca
cactggctcaccttcgggtgggcctttctgcgtttata

>Complete Sequence
XXXXXXXXXXXXXXXXXXXXXXXXXXXXXXXXXXXXXXXXagctgtcaccggatgtgctttccggtctgatgag
tccgtgaggacgaaacagcctctacaaataattttgtttaaACTAGA**AAAGAGGAGAAATACTAG**atgcgtaaaggc
gaagagctgttcactggtgtcgtccctattctggtggaactggatggtgatgtcaacggtcataagttttccgtgcgtggcgagggtgaag
gtgacgcaactaatggtaaactgacgctgaagttcatctgtactactggtaaactgccggtaccttggccgactctggtaacgacgctg
acttatggtgttcagtgctttgctcgttatccggaccatatgaagcagcatgacttcttcaagtccgccatgccggaaggctatgtgcagg
aacgcacgatttcctttaaggatgacggcacgtacaaaacgcgtgcggaagtgaaatttgaaggcgataccctggtaaaccgcattg
agctgaaaggcattgactttaaagaagacggcaatatcctgggccataagctggaatacaattttaacagccacaatgtgtacattac
cgcagataaacaaaaaaatggcattaaagcgaatttcaaaattcgccacaacgtggaggatggcagcgtgcagctggctgatcact
accagcaaaacactccaatcggtgatggtcctgttctgctgccagacaatcactatctgagcacgcaaagcgttctgtctaaagatccg
aacgagaaacgcgatcatatggttctgctggagttcgtaaccgcagcgggcatcacgcatggtatggatgaactgtacaaatgatga
ccaggcatcaaataaaacgaaaggctcagtcgaaagactgggcctttcgttttatctgttgtttgtcggtgaacgctctctactagagtca
cactggctcaccttcgggtgggcctttctgcgtttata

<u>No RiboJ Construct</u>

>Promoter Part
XXXXXXXXXXXXXXXXXXXXXXXXXXXXXXXXXXXXXXX

> B0034 w/ Spacer (from Lou *et al.* Supplement section V)
AAAGAGGAGAAATACTAG

>sfGFP (modified from Lou *et al.* Supplement section V)
**Atgcgtaaaggcgaagagctgttcactggtgtcgtccctattctggtggaactggatggtgatgtcaacggtcataagttttc
cgtgcgtggcgagggtgaaggtgacgcaactaatggtaaactgacgctgaagttcatctgtactactggtaaactgccggt
accttggccgactctggtaacgacgctgacttatggtgttcagtgctttgctcgttatccggaccatatgaagcagcatgactt
cttcaagtccgccatgccggaaggctatgtgcaggaacgcacgatttcctttaaggatgacggcacgtacaaaacgcgtg
cggaagtgaaatttgaaggcgataccctggtaaaccgcattgagctgaaaggcattgactttaaagaagacggcaatatcc
tgggccataagctggaatacaattttaacagccacaatgtgtacattaccgcagataaacaaaaaaatggcattaaagcga
atttcaaaattcgccacaacgtggaggatggcagcgtgcagctggctgatcactaccagcaaaacactccaatcggtgat
ggtcctgttctgctgccagacaatcactatctgagcacgcaaagcgttctgtctaaagatccgaacgagaaacgcgatcat
atggttctgctggagttcgtaaccgcagcgggcatcacgcatggtatggatgaactgtacaaatgatga**

>BBa_B0015 Part-only sequence – double terminator
**ccaggcatcaaataaaacgaaaggctcagtcgaaagactgggcctttcgttttatctgttgtttgtcggtgaacgctctctact
agagtcacactggctcaccttcgggtgggcctttctgcgtttata**

>Complete Sequence
XXXXXXXXXXXXXXXXXXXXXXXXXXXXXXXXXXXXXXX**AAAGAGGAGAAATACTAGatgcg
taaaggcgaagagctgttcactggtgtcgtccctattctggtggaactggatggtgatgtcaacggtcataagttttccgtgc
gtggcgagggtgaaggtgacgcaactaatggtaaactgacgctgaagttcatctgtactactggtaaactgccggtaccttg
gccgactctggtaacgacgctgacttatggtgttcagtgctttgctcgttatccggaccatatgaagcagcatgacttcttcaa
gtccgccatgccggaaggctatgtgcaggaacgcacgatttcctttaaggatgacggcacgtacaaaacgcgtgcggaa
gtgaaatttgaaggcgataccctggtaaaccgcattgagctgaaaggcattgactttaaagaagacggcaatatcctgggc
cataagctggaatacaattttaacagccacaatgtgtacattaccgcagataaacaaaaaaatggcattaaagcgaatttca
aaattcgccacaacgtggaggatggcagcgtgcagctggctgatcactaccagcaaaacactccaatcggtgatggtcct
gttctgctgccagacaatcactatctgagcacgcaaagcgttctgtctaaagatccgaacgagaaacgcgatcatatggttc
tgctggagttcgtaaccgcagcgggcatcacgcatggtatggatgaactgtacaaatgatgaccaggcatcaaataaaac
gaaaggctcagtcgaaagactgggcctttcgttttatctgttgtttgtcggtgaacgctctctactagagtcacactggctcac
cttcgggtgggcctttctgcgtttata**

The negative control plasmid consists of J23101 B0034 (as above) LacI (Bba_C0012 without LVA tail) and B0015 (as above).
tttacagctagctcagtcctaggtattatgctagcAAAGAGGAGAAATACTAGatggtgaatgtgaaaccagtaacgt
tatacgatgtcgcagagtatgccggtgtctcttatcagaccgtttcccgcgtggtgaaccaggccagccacgtttctgcgaaaacgcgg
gaaaaagtggaagcggcgatggcggagctgaattacattcccaaccgcgtggcacaacaactggcgggcaaacagtcgttgctga
ttggcgttgccacctccagtctggccctgcacgcgccgtcgcaaattgtcgcggcgattaaatctcgcgccgatcaactgggtgccagc
gtggtggtgtcgatggtagaacgaagcggcgtcgaagcctgtaaagcggcggtgcacaatcttctcgcgcaacgcgtcagtgggctg
atcattaactatccgctggatgaccaggatgccattgctgtggaagctgcctgcactaatgttccggcgttatttcttgatgtctctgaccag
acacccatcaacagtattattttctcccatgaagacggtacgcgactgggcgtggagcatctggtcgcattgggtcaccagcaaatcgc
gctgttagcgggcccattaagttctgtctcggcgcgtctgcgtctggctggctggcataaatatctcactcgcaatcaaattcagccgata
gcggaacgggaaggcgactggagtgccatgtccggttttcaacaaaccatgcaaatgctgaatgagggcatcgttcccactgcgatg
ctggttgccaacgatcagatggcgctgggcgcaatgcgcgccattaccgagtccgggctgcgcgttggtgcggatatctcggtagtgg
gatacgacgataccgaagacagctcatgttatatcccgccgttaaccaccatcaaacaggattttcgcctgctggggcaaaccagcgt
ggaccgcttgctgcaactctctcagggccaggcggtgaagggcaatcagctgttgcccgtctcactggtgaaaagaaaaaccaccct

ggcgcccaatacgcaaaccgcctctccccgcgcgttggccgattcattaatgcagctggcacgacaggtttcccgactggaaagcgg
gcagtga**ccaggcatcaaataaaacgaaaggctcagtcgaaagactgggcctttcgttttatctgttgtttgtcggtgaacgct
ctctactagagtcacactggctcaccttcgggtgggcctttctgcgtttata**
